# Fast-hyperspectral imaging remote sensing: Emission quantification of NO_2_ and SO_2_ from marine vessels

**DOI:** 10.1038/s41377-025-01922-x

**Published:** 2025-09-08

**Authors:** Chengzhi Xing, Shaocong Wei, Yikai Li, Peiyuan Jiao, Chao Liu, Jian Chen, Weiheng Wang, Haochen Peng, Yuhang Song, Cheng Liu

**Affiliations:** 1 Key Lab of Environmental Optics & Technology, Anhui Institute of Optics and Fine Mechanics, Hefei Institutes of Physical Science, Chinese Academy of Sciences, 230031 Hefei, China; 2 Department of Precision Machinery and Precision Instrumentation, University of Science and Technology of China, 230026 Hefei, China; 3 School of Environmental Science and Optoelectronic Technology, University of Science and Technology of China, 230026 Hefei, China; 4 Institute for Atmospheric and Climate Science, Eidgenössische Technische Hochschule Zürich, 8006 Zürich, Switzerland; 5 Center for Excellence in Regional Atmospheric Environment, Institute of Urban Environment, Chinese Academy of Sciences, 361021 Xiamen, China; 6 Key Laboratory of Precision Scientific Instrumentation of Anhui Higher Education Institutes, University of Science and Technology of China, 230026 Hefei, China

**Keywords:** Imaging and sensing, Atmospheric optics

## Abstract

Marine vessels play a vital role in the global economy; however, their negative impact on the marine atmospheric environment is a growing concern. Quantifying marine vessel emissions is an essential prerequisite for controlling these emissions and improving the marine atmospheric environment. Optical imaging remote sensing is a vital technique for quantifying marine vessel emissions. However, the available imaging techniques have suffered from insufficient detection accuracy and inadequate spatiotemporal resolution. Herein, we propose a fast-hyperspectral imaging remote sensing technique that achieved precise imaging of nitrogen dioxide (NO_2_) and sulfur dioxide (SO_2_) from marine vessels. Several key techniques are developed, including the coaxial design of three camera systems (hyperspectral camera, visible camera, and multiwavelength filters) and a high-precision temperature control system for a spectrometer (20 °C ± 0.5 °C). Moreover, based on the variation of O_4_ within them, plumes are categorized as aerosol-present and aerosol-absent, with different air mass factor (AMF) calculation schemes developed accordingly. Multiwavelength filters combined with spectral analysis enable precise identification of the plume outline and a detailed observation of the trace gas distribution inside the plume emitted from marine vessels. In addition, we focuse on the emission characteristics of NO_2_ and SO_2_ from large ocean cargo ships and small offshore cargo ships. Although there are still many emerging issues, such as measurement of cross-sections of trace gases at different temperature, nighttime imaging, and greenhouse gas imaging, this study opens a gate for synergies in pollution and carbon reductions and the continuous improvement of the marine atmospheric environment.

## Abstract (web summary)

A fast-hyperspectral imaging remote sensing technique was proposed to quantify nitrogen dioxide and sulfur dioxide emissions from marine vessels.

## Graphical abstract

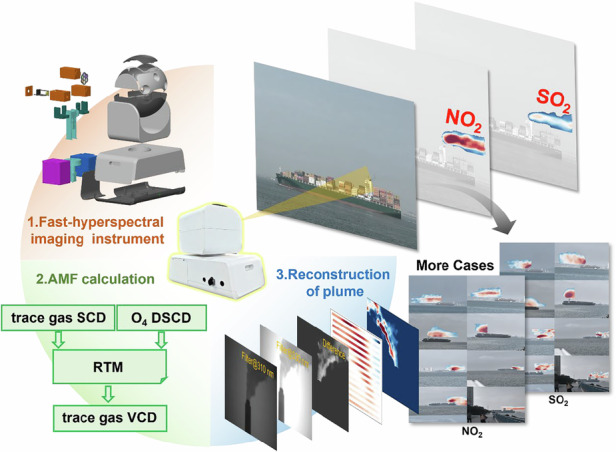

## Introduction

Ships play a vital role in the global economic system. Ships are the primary means of transportation for international trade and are responsible for transporting more than 80% of goods worldwide. However, the negative impact of ship emissions on the environment is a growing concern, especially in busy shipping channels and major port cities, where ship emissions have become one of the vital sources of air pollution^1–5^. Emissions from ships mainly include particulate matter, sulfur oxides (SO_x_), nitrogen oxides (NO_x_), and volatile organic compounds (VOCs), which not only pose a direct threat to the atmospheric environment but also have an indirect impact on the marine ecosystem^6–9^. With the growing global demand for environmental sustainability, the international community is paying increasing attention to the regulation of ship emissions. In this context, the development and application of efficient and accurate emission monitoring techniques are of importance.

Satellite and airborne remote sensing play a vital role in monitoring marine vessel emissions, revealing interannual variations and excessive emissions of pollutants, such as nitrogen dioxide (NO_2_) and sulfur dioxide (SO_2_), along with their corresponding impact on the coastal atmospheric environment^10–14^. However, the limited spatial and temporal resolution, cloud coverage, and other factors make it extremely challenging to extract ship emission signals from massive spectral data. These signals are significantly affected by the strong albedo of the sea surface as well as the spectral noise of the detector in the spatial dimension. These factors also impact the accuracy of satellite and airborne remote sensing, often resulting in an underestimation of the pollutant concentration from the target emission source. Portable measurement systems can be installed on ships, enabling continuous monitoring of pollution emissions from marine vessels. This method has the advantage of high accuracy but also entails high costs and maintenance requirements, and it is not possible to evaluate the diffusion of the ship’s plume in the atmosphere. In addition, long-path differential optical absorption spectroscopy (DOAS) and multiaxis DOAS are widely used for dynamic monitoring of ship emissions in inland waterways and harbors. However, the effectiveness of these monitoring techniques can be seriously affected by the overlapping of multiple ship emission plumes on the observation optical path and turbulent meteorological conditions^15,16^. Pollution emissions from different types of ships and under different operating conditions vary significantly, making the monitoring of pollution emission processes during ship voyages extremely relevant. The spectral imaging technique has significant advantages in pollution emission quantification and plume diffusion evaluation, which opens a window for assessing the impact of ship emissions on the marine and coastal atmospheric environment.

The current spectral imaging techniques for pollution monitoring include UV camera imaging, IR cold-screen camera imaging, Fourier infrared transform spectroscopic (FTIR) imaging, and UV–visible spectroscopic imaging^17–29^. However, the disadvantages of these imaging techniques are relatively apparent. UV cameras and IR cold-screen cameras, using fixed wavelengths, are susceptible to interference from the absorption of other trace gases at the same wavelengths. Moreover, these cameras can only characterize whether emissions occurred or if there was a change in emission intensity but cannot quantify the emitted species and their corresponding concentrations. FTIR can image the emission plumes of various trace gases, especially VOCs. However, it is limited by its high detection limit and inability to measure reactive nitrogen oxides (e.g., HONO), which are vital contributors to atmospheric oxidation capacity. This limitation makes it difficult to precisely quantify and evaluate the impacts of emitted pollutants on the atmospheric environment and human health. In addition, due to the stability requirements of Michelson interferometry, FTIR struggles with mobile imaging of multiple emission sources. The UV–visible spectroscopic imaging technique can be divided into line array CCD imaging and plane array CCD imaging. Plane array CCD imaging has the technical advantage of high spatiotemporal resolution, but its stripe effect seriously limits the number of detectable components. This is mainly because the absorption of VOCs in the UV band is primarily at 320 nm. Under the combined effects of the low spectral signal-to-noise ratio, the weak absorption characteristic of VOCs, and the cross-absorption of multiple VOCs, the absorption signals of VOCs are easily submerged in the stripe noise. The line array CCD imaging technique can avoid the influence of the stripe effect to a certain extent. However, the observation scheme of grid-by-grid spectral acquisition significantly reduce the imaging spatiotemporal resolution and increased the difficulty of plume identification. Therefore, the development of a hyperspectral imaging technique for air pollution emissions with high accuracy and spatiotemporal resolution is necessary and urgent.

Herein, we report the design and implementation of a fast-hyperspectral imaging technique to achieve high-precision and high spatiotemporal resolution imaging of NO_2_ and SO_2_ emitted from marine vessels. A series of key challenges, such as large spectral noise and the difficulty in fine plume identification, have been overcome.

## Results

### Instrument design and observation scheme

**Fig. 1:**
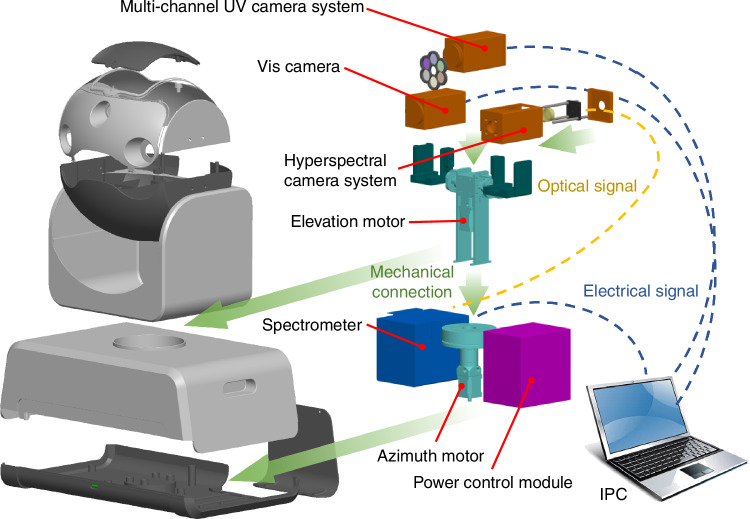
Fast-hyperspectral imaging remote sensing instrument

**Fig. 2:**
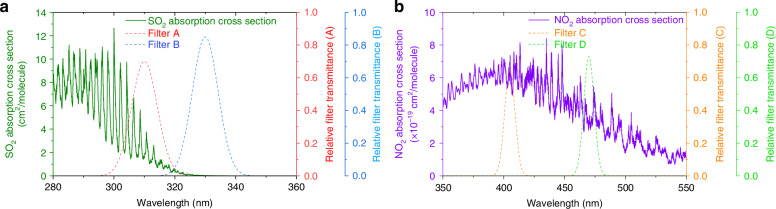
SO_2_ and NO_2_ detection using filters. **a** SO_2_ absorption cross-section and transmission curves of filters A and B. **b** NO_2_ absorption cross-section and transmission curves of filters C and D

The fast-hyperspectral imaging remote sensing instrument consists of six major parts: a visible camera, a multi-channel UV camera system, a hyperspectral camera system, a 2D scanning system, a power control module, and an industrial control machine (IPC) (Fig. 1). The visible camera is used to record live images of the imaging area. The multi-channel UV camera system consists of a UV camera and a filter wheel, with an advantage of high imaging spatial resolution to help to identify plume contours and the absorption intensity of pollutants inside the plume. The filter wheel is equipped with several pairs of filters, each designed for a specific pollutant gas, with their central wavelengths corresponding to the strong and weak absorption bands of that gas. The different UV images passing through each pair of filters will be collected by the UV camera and stored in the IPC. As shown in Fig. 2a, we select two filters with center wavelengths of 310 and 330 nm, corresponding to relative filter transmittances of 0.7 and 0.85, respectively, to detect SO_2_. Two filters with center wavelengths of 405 and 470 nm, corresponding to relative filter transmittances of 0.65 and 0.73, are selected to detect NO_2_ (Fig. 2b). The hyperspectral camera system consists of a telescope a fiber and a spectrometer, with an advantage of high quantification accuracy of pollutants. It is used to collect solar scattering spectra, which are converged by the quartz lens to the entrance of the multimode fiber and then transmitted to the spectrometer through the fiber. The field of view (FOV) of the hyperspectral camera is detected with the aid of an illuminant (Supplement sect. S1). The 2D scanning system consists of an elevation motor and an azimuth motor to control the elevation and horizontal rotation of the telescope, respectively. The power control module provides stable power to the components of the instrument, and the IPC is used to run the upper computer software, which controls the operation of the instrument and analyzes the spectral results. The main parameters of the fast-hyperspectral imaging remote sensing instrument are shown in Table 1.

**Table 1:** Major parameters of the fast-hyperspectral imaging remote sensing instrument

Modules	Parameters
Visible camera	Resolution: 2560 × 1440 px
UV camera	Wavelength range: 200–900 nmQuantum efficiency: 56% @310 nm, 60% @330 nm, 81% @405 nm, 97% @470 nm
Filters	310, 330, 405, and 470 nm
Hyperspectral camera	FOV: <0.5°, scattered light transmittance: >75%
Elevation motor	Angles: −5°–90°, precision: <0.1°
Azimuth motor	Angles: 0°–180°, precision: <0.1°
Spectrometer	Wavelength range: 300–400 nm, resolution: 0.46 nm

**Fig. 3:**
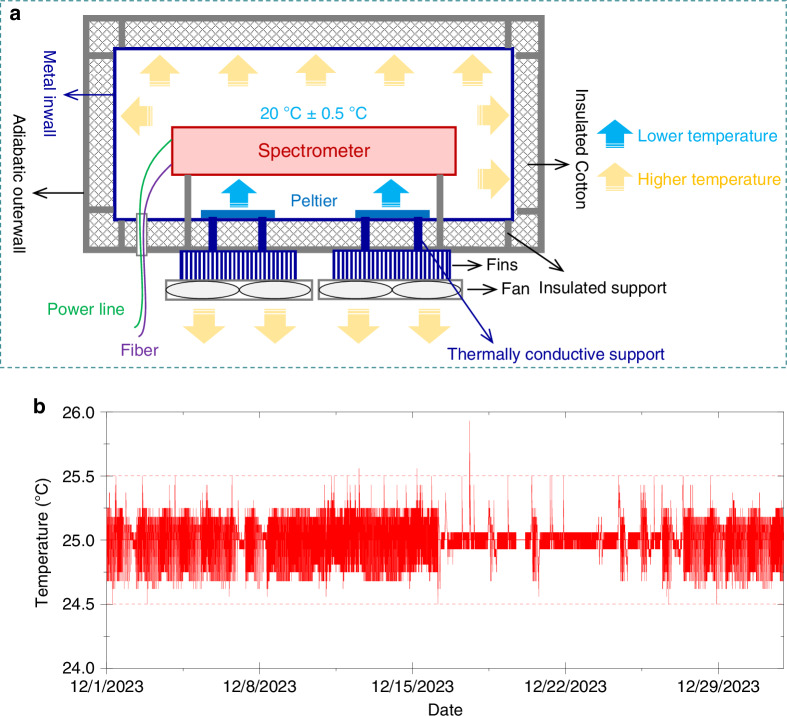
Temperature control for the spectrometer. **a** Temperature control system for the spectrometer. **b** Temperature fluctuation in the thermostatic convection chamber

To reduce the noise of the spectrometer, we propose a temperature control system. As shown in Fig. 3, the main body of this temperature control system is a thermostatic convection chamber, which has an adiabatic outerwall and a metal inwall filled with insulated cotton to prevent the intrusion of external heat. As soon as the temperature inside the chamber fluctuates, as detected by temperature sensors (pt100), the Peltiers (up to 60 W × 2) will be activated for cooling/heating to maintain the inside temperature at 20 °C ± 0.5 °C. The excess heat is directed out of the chamber through fins and fans. The stability test of this system under different temperature conditions is carried out (Fig. S2).

During automatic imaging, the IPC controls the elevation and azimuth angles of the telescope through the 2D scanning system according to preset instructions to achieve continuous scanning in an “S”-shaped trajectory, covering the entire preset imaging area (Fig. S3). The direct spectral analysis results are differential slant column densities (DSCDs) of NO_2_ and SO_2_. The scanning and spectra collection are simultaneous, and the integration time of a single spectrum is 3 s. Two zenith measurements should be conducted before the serpentine scanning as reference spectra for the entire observation. A complete scanning process for a plume generally takes <4 min, and the imaging spatial resolution is <0.5 m × 0.5 m, which depends on the FOV of the hyperspectral camera and the distance between the instrument and the emission source (Fig. S4).

### Air mass factor calculation

The calculation accuracy of vertical column density (VCD) depends on air mass factor (AMF), which is mainly affected by the stereoscopic distribution of aerosols. Variations in atmospheric radiative transfer due to aerosols inside the plume can cause significant changes in O_4_ DSCDs^30^. Therefore, we select a fixed elevation angle passing through the plume and indirectly determined the presence of aerosols within the plume by analyzing the variation of O_4_ DSCDs at different azimuth angles. Considering the influence of air mass transport in the surrounding atmosphere and the cloud effect, we define that aerosols are absent inside the plume when the standard deviation of the O_4_ DSCDs at different azimuth angles was <20%.

The AMF within the plume is divided into two scenarios for calculation: with and without aerosols. If there are no aerosols within the plume, the aerosol vertical profiles at different azimuth angles should be retrieved and input as constraints into the radiative transfer model (RTM) to obtain AMFs at different azimuths. If aerosols are present within the plume, the stereoscopic distribution of aerosols within the plume needs to be simulated and reconstructed with the help of 3D-RTM. This process is supported by the optical estimation method (OEM)^31^. When the simulated O_4_ DSCDs and the observed O_4_ DSCDs are in perfect agreement, the simulated aerosol stereoscopic distribution is considered to be the true aerosol stereoscopic distribution within the plume and is used to calculate the AMF.

### Reconstruction of plume

**Fig. 4:**
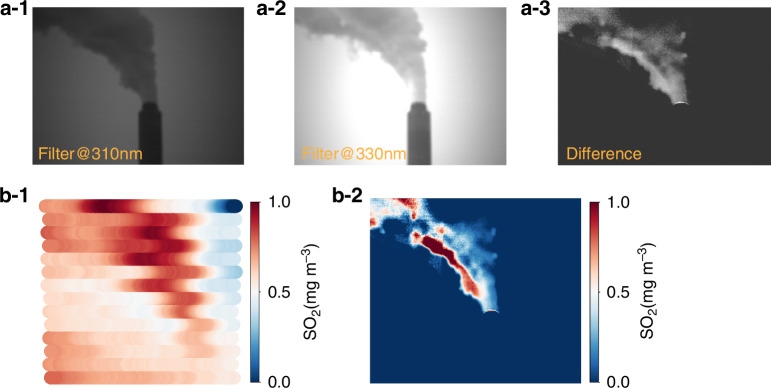
Reconstruction of plume trace gas concentrations. **a-1** Plume signals collected through the filter @310 nm. **a-2** Plume signals collected through the filter @330. **a-3** Difference in signals between filters @310 nm and @330 nm. **b-1** SO_2_ concentrations in the plume measured by the hyperspectral camera. **b-2** Reconstruction of the plume with SO_2_ distributions

Uncertainty in the plume range, limited by imaging spatial resolution, can seriously affect the accurate evaluation of trace gas emission flux^29^. To precisely identify the plume outline and quantify the concentrations of trace gases within plumes, high-resolution imaging techniques are essential. A plume reconstruction scheme is proposed. The flowchart of plume reconstruction is shown in Fig. S5. SO_2_ is selected as a typical trace gas emitted from a coal-fired power plant to demonstrate the plume reconstruction process (Fig. 4). As an example, two filters with central wavelengths of 310 and 330 nm are selected for SO_2_. Figure 4a1–2 shows the corresponding signals collected using these two filters. The absorption of the optical signal by SO_2_ at 310 nm is more significant than that at 330 nm. The difference in absorption intensities between these two filters is shown in Fig. 4a3, which characterizes the relative light intensity absorbed by SO_2_ within the plume, corresponding to different levels of SO_2_. Figure 4b-1 shows the SO_2_ imaging results captured by the hyperspectral camera. The SO_2_ plume is observed, but the plume outline is vague and the SO_2_ distribution within the plume is not sufficiently detailed. Therefore, SO_2_ distribution weights within the plume are established based on its differential absorption intensities at 310 and 330 nm, and these absorption intensities within the plume are normalized. The spatial resolution depends on the pixel size of the filter camera. By correcting the results shown in Fig. 4b-1 using the normalized SO_2_ distribution weights within the plume, the true SO_2_ plume with high spatial resolution can be obtained. In Fig. 4b-2, we find that the maximum SO_2_ concentration within the plume reached 0.94 mg m^−3^. However, this maximum concentration does not occur at the outlet, which can be attributed to variations in coal properties during emissions and SO_2_ accumulations caused by changing meteorological conditions. Additional representative imaging examples are provided in Fig. S6. Compared to previous imaging techniques (e.g., Imaging DOAS), the fast-hyperspectral imaging remote sensing instrument achieves precise quantification of pollutants while delivering higher spatial resolution, enabling detailed characterization of pollution plume structures (Fig. S7).

### NO_2_ and SO_2_ emission imaging from marine vessels

**Fig. 5:**
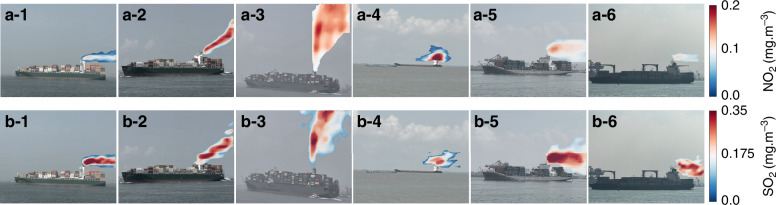
NO_2_ and SO_2_ imaging results from the plume of marine vessels. **a-1** – **a-3** NO_2_ plume imaging from large ocean cargo ships. **b-1** – **b3** SO_2_ plume imaging from large ocean cargo ships. **a-4** – **a-6** NO_2_ plume imaging from offshore small cargo ships. **b-4** ~ **b6** SO_2_ plume imaging from offshore small cargo ships

**Fig. 6:**
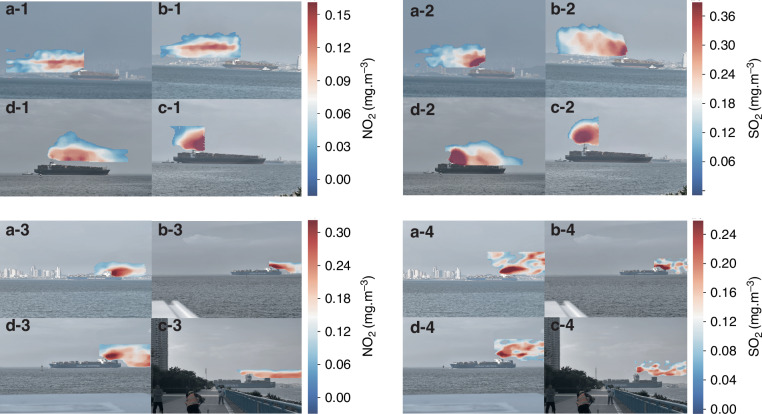
NO_2_ and SO_2_ plume imaging from large ocean cargo ships at different distances. **a** The ship is 1000 m from the harbor. **b** The ship is 600 m from the harbor. **c** The ship is 500 m from the harbor. **d** The ship is 300 m from the harbor

Figure 5 shows the NO_2_ and SO_2_ emission imaging results from the large ocean cargo ships and offshore small cargo ships in Qingdao. The maximum concentrations of NO_2_ from the large ocean cargo ships are 0.124 ± 0.011, 0.201 ± 0.015, and 0.174 ± 0.014 mg m^−3^, respectively, whereas the maximum concentrations of SO_2_ are 0.425 ± 0.034, 0.372 ± 0.029, and0.342 ± 0.027 mg m^−3^, respectively. The maximum concentrations of NO_2_ from the offshore small cargo ships are 0.251 ± 0.023, 0.148 ± 0.012, and 0.134 ± 0.011 mg m^−3^, respectively, whereas the maximum concentrations of SO_2_ are 0.334 ± 0.027, 0.389 ± 0.032, and 0.397 ± 0.031 mg m^−3^, respectively. As shown in Fig. 6, we can find significant differences in the maximum emission concentrations of NO_2_ and SO_2_ from large ocean cargo ships at different distances from the port. For the first large ocean cargo ship, the maximum concentrations of NO_2_ emitted from the large ocean cargo ship at 1,000, 600, 500, and 300 m from the port are 0.137 ± 0.012, 0.139 ± 0.012, 0.155 ± 0.014, and 0.144 ± 0.013 mg m^−3^, respectively (Fig. 6a-1–d-1). The maximum concentrations of SO_2_ emitted from the large ocean cargo ship at 1,000, 600, 500, and 300 m from the port are 0.461 ± 0.041, 0.458 ± 0.037, 0.487 ± 0.039, and 0.482 ± 0.038 mg m^−3^, respectively (Fig. 5a-2–d-2). For the second large ocean cargo ship, the maximum concentrations of NO_2_ emitted from the large ocean cargo ship at 1,000, 600, 500, and 300 m from the port are 0.315 ± 0.027, 0.334 ± 0.028, 0.194 ± 0.017, and 0.321 ± 0.029 mg m^−3^, respectively (Fig. 6a-3–d-3). The maximum concentrations of SO_2_ emitted from the large ocean cargo ship at 1,000, 600, 500, and 300 m from the port are 0.295 ± 0.024, 0.311 ± 0.028, 0.235 ± 0.020, and 0.324 ± 0.028 mg m^−3^, respectively (Fig. 5a-4–d-4).

The emission fluxes of NO_2_ from the large ocean cargo ships are 1.57 ± 0.17, 3.11 ± 0.29, and 3.42 ± 0.34 kg/h, respectively. However, the emission fluxes of SO_2_ from the large ocean cargo ships are 2.65 ± 0.31, 2.71 ± 0.35 and 2.77 ± 0.27 kg/h, respectively. The emission fluxes of NO_2_ from the offshore small cargo ships are 2.75 ± 0.31, 0.93 ± 0.11, and 0.78 ± 0.08 kg/h, respectively. However, the emission fluxes of SO_2_ from the large ocean cargo ships are 1.01 ± 0.11, 1.85 ± 0.21 and 1.34 ± 0.17 kg/h, respectively. The higher SO_2_ emission flux from the large ocean cargo ship compared to the offshore small cargo ship is due to the large ocean cargo ship using heavy oil. For the first large ocean cargo ship, the NO_2_ emission fluxes from the large ocean cargo ship at 1,000, 600, 500, and 300 m from the port are 0.33 ± 0.03, 0.37 ± 0.04, 0.25 ± 0.02, and 0.41 ± 0.05 kg/h, respectively (Fig. 6a-1–d-1). However, the SO_2_ emission fluxes from the large ocean cargo ship at 1,000, 600, 500, and 300 m from the port are 1.17 ± 0.14, 1.05 ± 0.12, 0.79 ± 0.09, and 0.45 ± 0.06 kg/h, respectively (Fig. 6a-2–d-2). For the second large ocean cargo ship, the NO_2_ emission fluxes from the large ocean cargo ship at 1,000, 600, 500, and 300 m from the port are 0.67 ± 0.07, 0.74 ± 0.07, 0.35 ± 0.04, and 0.77 ± 0.10 kg/h, respectively (Fig. 6a-3–d-3). However, the SO_2_ emission fluxes from the large ocean cargo ship at 1,000, 600, 500, and 300 m from the port are 0.97 ± 0.12, 0.95 ± 0.12, 0.74 ± 0.08, and 1.02 ± 0.14 kg/h, respectively (Fig. 6a-2–d-2). This should be attributed to the use of high-quality fuel and the reduced power of the large cargo ship as it approaches the port.

## Discussion

This hyperspectral imaging technique proposes another idea for monitoring and management of the atmospheric environment. Moreover, emission inventory provides relevant technical support for emission quantification, model development, and pollution control. The current emission inventories are primarily established based on measurement and emission factor estimation methods^32–34^. However, these inventories have a significant timeliness issue and cannot be updated in real time for some newly emerged emission sources or emission sources with significant changes in emission characteristics. The current emission inventories are usually coupled with meteorochemical models to simulate the emission characteristics of trace gases and the corresponding various physicochemical processes, enabling the spatiotemporal dynamization of inventories^35–37^. Uncertainties in inventories are mainly due to model uncertainties. Additionally, the uncertainty of discontinuous measurements and source-specific measurements on the development of dynamic emission inventory is mainly due to the inadequate evaluation of plume dispersion. The hyperspectral imaging technique will open a window for the establishment of dynamic emission inventories driven by measurements of different types of sources. Certainly, the large-scale implementation of hyperspectral imaging observations combined with deep learning models has enormous potential for establishing a dynamic emission inventory of trace gases at different regional scales.

In addition, a vital factor affecting the accuracy of hyperspectral imaging is the absorption cross-sections of trace gases. However, the issues of incomplete species, insufficient spectral resolution, and insufficient temperature/pressure gradients in the available absorption cross-section libraries of trace gases are significant. It is necessary to develop equipment that can stabilize cross-sections with high spectral resolution and multiple temperature/pressure gradients of various trace gases in the future. The main difficulties of this technique mainly include the phase state control of target substances, the absorption inhibition of the inwall of the absorption cavity, the stable control of temperature and pressure in the absorption cavity, and the enhancement of the optical path.

Nighttime atmospheric physicochemical processes play an extremely vital role in the overall atmospheric system. However, the above hyperspectral imaging technique, which used scattered sunlight as the light source, can not monitor emissions at night. In view of this, we will propose a future-proof technique for nighttime hyperspectral imaging of trace gas emissions. As shown in Fig. S8, this system will mainly consists of four parts: hyperspectral active instrument, high-precision tracking module, UAV reflector module, and spectroscopy and detection module. The light source of the active instrument will be a multiwavelength-coupled LED, which has a sufficient wavelength range and luminous power. To prevent wavelength drift of LEDs due to temperature variations, precise temperature control of each LED will be required. The reflector array should be mounted on a UAV platform and configured with a gimbal to prevent irregular jittering. Moreover, the high-precision tracking module will modulate the light beam according to the motion information of the UAV to achieve dynamic tracking of the beam on the reflector array. Subsequently, the absorbed broadband spectra will be reflected to the receiving module of the hyperspectral active instrument in real time. The exposure time of each spectrum should be at least 3 s. This technique will allow for accurate imaging of nighttime emissions of trace gases if the flight path of the UAV can be preplanned.

Finally, with China’s proposal of “Carbon Peak and Carbon Neutral”^38–40^, carbon monitoring and carbon emission reduction have been given high priority. Accurate spectroscopic imaging techniques for greenhouse gases need to be urgently emphasized and developed. Fourier transform spectroscopy and grating spectroscopy will still be two of the most vitally dependent techniques, but grating spectroscopy faces more challenges in achieving spectral resolution ( ≤ 0.1 nm).

## Materials and methods

### Spectral analysis

**Fig. 7:**
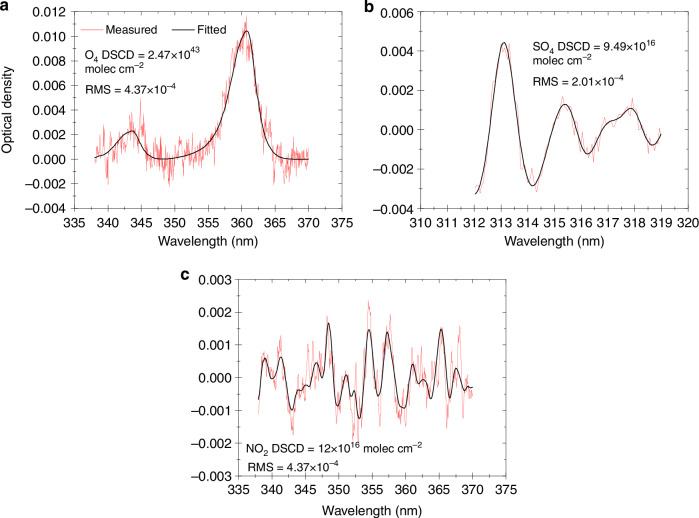
Typical spectral fitting results. **a**–**c** O_4_, SO_2_ and NO_2_ fitting examples. The black and red curves indicate the fitted absorption structures of target species and the derived absorption structures of target species from the measured spectra plus the fit residual, respectively

Spectral analysis is based on Lambert–Beer law. The zenith spectrum is used as the reference spectrum and differed from the off-axis observed spectrum. The DSCDs of O_4_, NO_2_, and SO_2_ are calculated using the QDOAS software developed through the least squares algorithm (https://uv-vis.aeronomie.be/software/QDOAS/LastChanges.php). The detailed retrieval settings for these species are listed in Table 2. The typical spectral fitting results are shown in Fig. 7 and S9. Moreover, the fitting results with root mean squares larger than 5.0 × 10^−4^ for O_4_ and NO_2_ and 6.0 × 10^−4^ for SO_2_ are filtered out to ensure data quality.

**Table 2:** Detailed retrieval settings of O_4_, NO_2_, and SO_2_

Parameter	Data source	Fitting intervals (nm)		
		O_4_	NO_2_	SO_2_
Wavelength range		338–370	338–370	307.5–315
NO_2_	298 K, I_0_-corrected*, Vandaele et al.^44^	√	√	√
NO_2_	220 K, I_0_-corrected*, Vandaele et al.^44^	√	√	×
O_3_	223 K, I_0_-corrected*, Serdyuchenko et al.^45^	√	√	√
O_3_	243 K, I_0_-corrected*, Serdyuchenko et al.^45^	√	√	×
O_4_	293 K, Thalman and Volkamer^46^	√	√	×
SO_2_	298 K, Vandaele et al.^47^	×	×	√
H_2_O	HITEMP (Rothman et al. 2010)^48^	√	√	×
BrO	223 K, Fleischmann et al.^49^	√	√	×
HCHO	298 K, Meller and Moortgat^50^	√	√	√
Ring	Calculated with QDOAS	√	√	√
Polynomial degree		Order 3	Order 3	Order 5
Intensity offset		Constant	Constant	Order 1

*Solar I_0_ correction by Aliwell et al.^51^

### Aerosol vertical profile retrieval

The aerosol vertical profiles are retrieved using OEM^31^, which employed the RTM VLIDORT^41^ as the forward model. The state vector *x* is determined when the following cost function ${\chi}^2$ reached a minimum value.

$$\chi^{2}(x)=[y-F(x,b)]^{T} S_{\in}^{-1}[y-F(x,b)]+[x-x_{a}]^{T}S_{a}^{-1}[x-x_{a}]$$

$F (x,b)$, the forward model, describes the measurement vector *y* (measured O_4_ DSCDs) as a function of the atmospheric state vector *x* (aerosol vertical profiles) and other atmospheric parameters *b* (aerosol optical properties, pressure, and temperature). *x*_*a*_ represents the a priori vector of aerosol. $S_\epsilon$ and $S_a$ represent the covariance matrices of *y* and *x*_*a*_, respectively. During retrieval, Jacobians of $F (x,b)$ are used as weighting functions to describe the variations of simulated O_4_ DSCDs when *x* is changed^42^. We set a 0.1-km vertical resolution for aerosol profiles under 2.0 km, and the single scattering albedo is calculated based on multiple-wavelength O_4_ absorptions^43^. The error sources of aerosol can be divided into smoothing and noise errors, algorithm errors, and cross-sectional errors, and the detailed aerosol error analysis is described in Supplement Sect. S2, and the aerosol error is at 10–16% in this study.

### Quantification of imaging concentrations and emission fluxes of NO_2_ and SO_2_

The flowchart of NO_2_ and SO_2_ concentration calculation is depicted in Fig. S10. The VCD is calculated using the formula VCD = DSCD/DAMF, where DAMF is differential air mass factor (Supplement sect. S3). The conversion process from VCD to mass concentration (*c*: μg/m3) is described using following equation^29^.

$${c}_{i,j} (k \,{\cdot}\, M{\cdot} {VCD}_{i,j})/({h}_{i,j}\,{\cdot}\,N_A)$$

Where, *i* and *j* represent the indexes of elevation and azimuth angles. *M* and *N*_*A*_ represent the relative molecular mass (g/mol) and Avogadro’s constant (mol^−1^), respectively. *h* represents the calculated height (m). *k* represents the unit conversion factor, which equals 1.0 × 10^10^. To verify the accuracy of the fast-hyperspectral imaging remote sensing system, we select two accuracy calibration and verification methods, a laboratory calibration method to observe a standard gas with a known concentration gradient, and an outdoor verification method to compare the imaging and online in situ measurements (Fig. S11). Moreover, the imaging error analysis is shown in Supplement Sect. S4.

To further evaluate the emissions from marine vessels, we calculate the emission fluxes of NO_2_ and SO_2_ using the following equation. They depend on the cross-section of the plume, the concentrations of NO_2_ and SO_2_ in the plume and the wind speed perpendicular to the cross-section of the plume (Fig. S12). The uncertainties in NO_2_ and SO_2_ emission fluxes arise from the spectral analysis, air mass factor, unit conversion, plume cross-section and wind speed (Supplement Sect. S4 and S5).

$$Flux=\frac{c_{j}\cdot (\pi\cdot r^{2})\cdot \Delta x\sin \gamma}{\Delta t}=c_{j}\cdot (\pi\cdot r^{2})\cdot v_{wind}\cdot \sin\gamma$$

Where, *c*_*j*_ represents the average concentration of the target trace gas observed at azimuth *j*. *r*, *v*_*wind*_, and *γ* denote plume radius, wind speed, and the angle between the observation azimuth and wind direction, respectively.

## Supplementary information

Supplementary Information for Fast-hyperspectral imaging remote sensing: Emission quantification of NO2 and SO2 from marine vessels

## Data Availability

The NO_2_ and SO_2_ imaging data are available from Chengzhi Xing (xingcz@aiofm.ac.cn) and Cheng Liu (chliu81@ustc.edu.cn) upon reasonable request.
