# Supplementary material for: Fast-hyperspectral imaging remote sensing: Emission quantification of NO2 and SO2 from marine vessels
Source: Light Sci Appl. 2025 Sep 8;14:308. doi: 10.1038/s41377-025-01922-x (PMC12420823; doi:10.1038/s41377-025-01922-x)
Supplement: Supplementary file 1 — Supplementary Information for Fast-hyperspectral imaging remote sensing: Emission quantification of NO2 and SO2 from marine vessels [file 41377_2025_1922_MOESM1_ESM.docx]

*Supplementary Information for*

Fast-hyperspectral imaging remote sensing: Emission quantification of NO_2_ and SO_2_ from marine vessels

Chengzhi Xing^1^, Shaocong Wei^2^, Yikai Li^3^, Peiyuan Jiao^2^, Chao Liu^1,2^, Jian Chen^2^, Weiheng Wang^2^, Haochen Peng^1,2^, Yuhang Song^4^, Cheng Liu^1,2,5,6,^*

^1^Key Lab of Environmental Optics & Technology, Anhui Institute of Optics and Fine Mechanics, Hefei Institutes of Physical Science, Chinese Academy of Sciences, 230031, Hefei, China

^2^Department of Precision Machinery and Precision Instrumentation, University of Science and Technology of China, 230026, Hefei, China

^3^School of Environmental Science and Optoelectronic Technology, University of Science and Technology of China, 230026, Hefei, China

^4^Institute for Atmospheric and Climate Science, Eidgenössische Technische Hochschule Zürich, 8006, Zürich, Switzerland

^5^Center for Excellence in Regional Atmospheric Environment, Institute of Urban Environment, Chinese Academy of Sciences, 361021, Xiamen, China

^6^Key Laboratory of Precision Scientific Instrumentation of Anhui Higher Education Institutes, University of Science and Technology of China, 230026, Hefei, China

*Correspondence: [chliu81@ustc.edu.cn](mailto:chliu81@ustc.edu.cn) (C.L.)

**Section S1. The field of view (FOV) of the hyperspectral camera**

As shown in Fig. S1, β was true field of the illuminant, which was known. The value of α was determined using 2α+β through rotating the hyperspectral camera when light intensity was less than 10% of the maximum detected light intensity. Moreover, to ensure that hyperspectral camera and visible camera were absolutely coaxial, the angle () between them was corrected using following equation.

Where *θ* was the FOV of visible camera. (*a, b*) were the horizontal and vertical pixels of the target area array. (*x, y*) and (*x’, y’*) were the pixel coordinates of the center of the FOV of hyperspectral camera and visible camera during observation, respectively.

**Section S2. The error analysis of retrieved atmospheric aerosol**

For aerosol profile retrieved results, we conduct the error analysis on aerosol optical density (AOD), and near-surface (0-100 m) aerosol extinction coefficients (AECs). The error sources considered are listed below.

1. Smoothing and noise errors refers to the fitting error of DOAS fits. By calculating the averaged error of retrieved profiles, we obtained smoothing and noise errors on near surface AECs and AOD, which are 12% and 5%, respectively.
2. Algorithmic error points to an imperfect minimum of the cost function, i.e., the difference between measured and modeled O_4_ dSCDs. Based on the fact that measurements at 5^o^ and 30^o^ elevation angles are sensitive to the lower and upper air layers, respectively, the average relative differences between measured and modeled O_4_ dSCDs for a 5^o^ and 30^o^ elevation angle are usually utilized to estimate the algorithm error on near surface AECs and AOD, respectively. Notably, algorithm error cannot be realistically estimated because it is difficult to assign the differences between measured and modeled O_4_ dSCDs to each height of the aerosol profile. Considering its trivial role in the total error budget, we estimated these errors on the near surface AECs and AOD at 3% and 6%, respectively, according to Wang et al. (2017).
3. Cross section error is 4% for O_4_ (aerosol) (Thalman and Volkamer, 2013).
4. The errors related to the temperature dependence of the cross sections can be estimated as follows. With two cross sections at two temperatures, we firstly calculated the amplitude changes of the cross sections per kelvin. Subsequently, we multiplied this with the variation magnitude of the surrounding temperature. The corresponding errors of O_4_ (aerosols) is around 10%.

The total error is calculated by combining all the error terms in Gaussian error propagation, which are 16% and 13% for the near surface AECs and AOD, respectively. The smoothing and noise error plays a dominant role in the total error estimation of the near surface AECs and AOD.

**Section S3. The calculation of vertical column density (VCD)**

The vertical column density (VCD) was calculated referring to the following equation.

$$VCD=\frac{DSCD}{DAMF}=\frac{SCD_{\alpha}-SCD_{zenith}}{AMF_{\alpha}-AMF_{zenith}}$$

Where $\alpha$ indicated the off-zenith elevation angles.

**Section S4. The error analysis of concentration imaging and flux calculation**

The imaging errors of NO_2_ and SO_2_ concentrations depended on the processes of spectral analysis, air mass factor (AMF) calculation and unit conversion (Xing et al., 2024). The uncertainties in NO_2_ and SO_2_ emission fluxes, in addition to the errors mentioned above, also arises from the uncertainty in the plume cross-section and the uncertainty in wind speed.

The uncertainty in the plume cross-section (*e_plume_*) depended on the imaging spatial resolution, and it can be describe using the following equation.


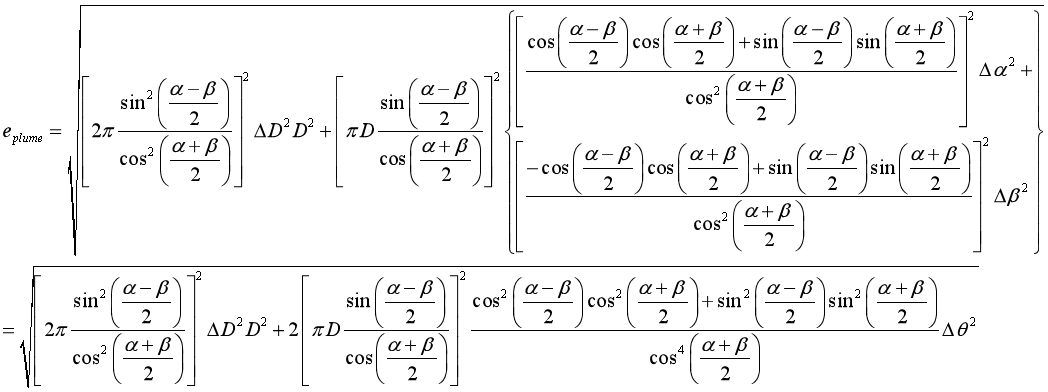


Where, $\Delta\alpha=\Delta\beta=\Delta\theta$ is half of the height of the imaging grid. *D* is the distance between the instrument and the imaging marine vessels.

The wind speed and direction were simulated by Weather Research and Forecasting (WRF) model. The detailed model description and its physical parameterization scheme can be found in Zhu et al. (2021). The horizontal resolution was 1.0 × 1.0 km^2^, and 44 vertical layers were set from the ground surface to 5000 Pa. The uncertainty of wind speed (*e_speed_*) depended on the uncertainty in the WRF model simulations and can be described using the following equation.

$$e_{speed}=\sqrt{\overline{Cov(u,v)}+\mathrm{var}(u_{1}-u_{0})+\mathrm{var}(v_{1}-v_{0})}$$

Where, $(u_{0},v_{0})$ is the measured wind field at a meteorological station. $(u_{1},v_{1})$ is the WRF simulated wind field at the meteorological station. $\overline{Cov(u,v)}$ is the average covariance of the wind field within a circular effective area centered at the imaging point, with a radius of 2D.

**Section S5. The determination of cross section of the plume and the calculation of the emission fluxes of NO_2_ and SO_2_**

When the concentration in high-value grid was more than twice that of in adjacent low-value grid at one azimuth angle, it can be considered that these two grids constitute the boundary of the plume. The emission fluxes of NO_2_ and SO_2_ depends on the cross-section of the plume, the concentrations of NO_2_ and SO_2_ in the plume and the wind speed perpendicular to the cross-section of the plume. Figure S12 showed a theoretical diagram of the determination of cross section of the plume and the calculation of the emission fluxes of NO_2_ and SO_2_.


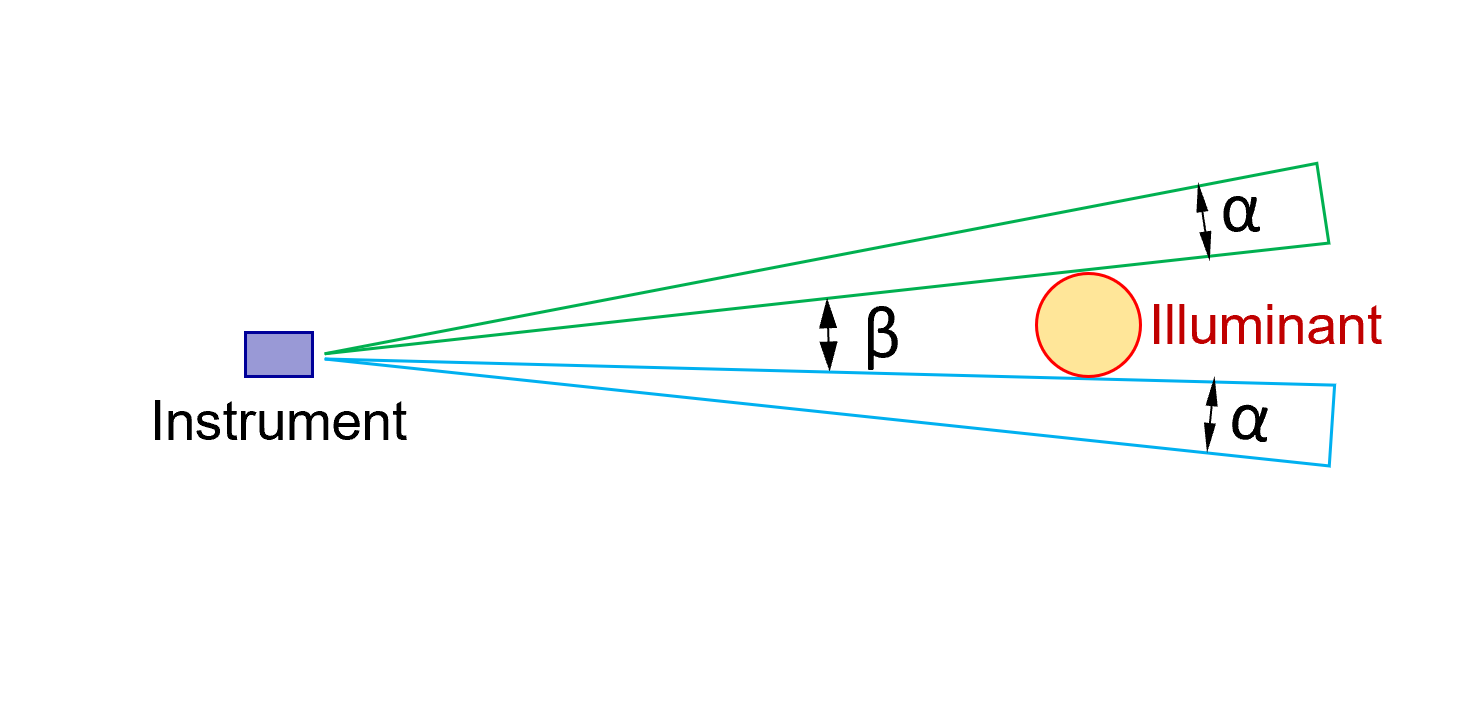


Fig. S1. Field of view calibration.


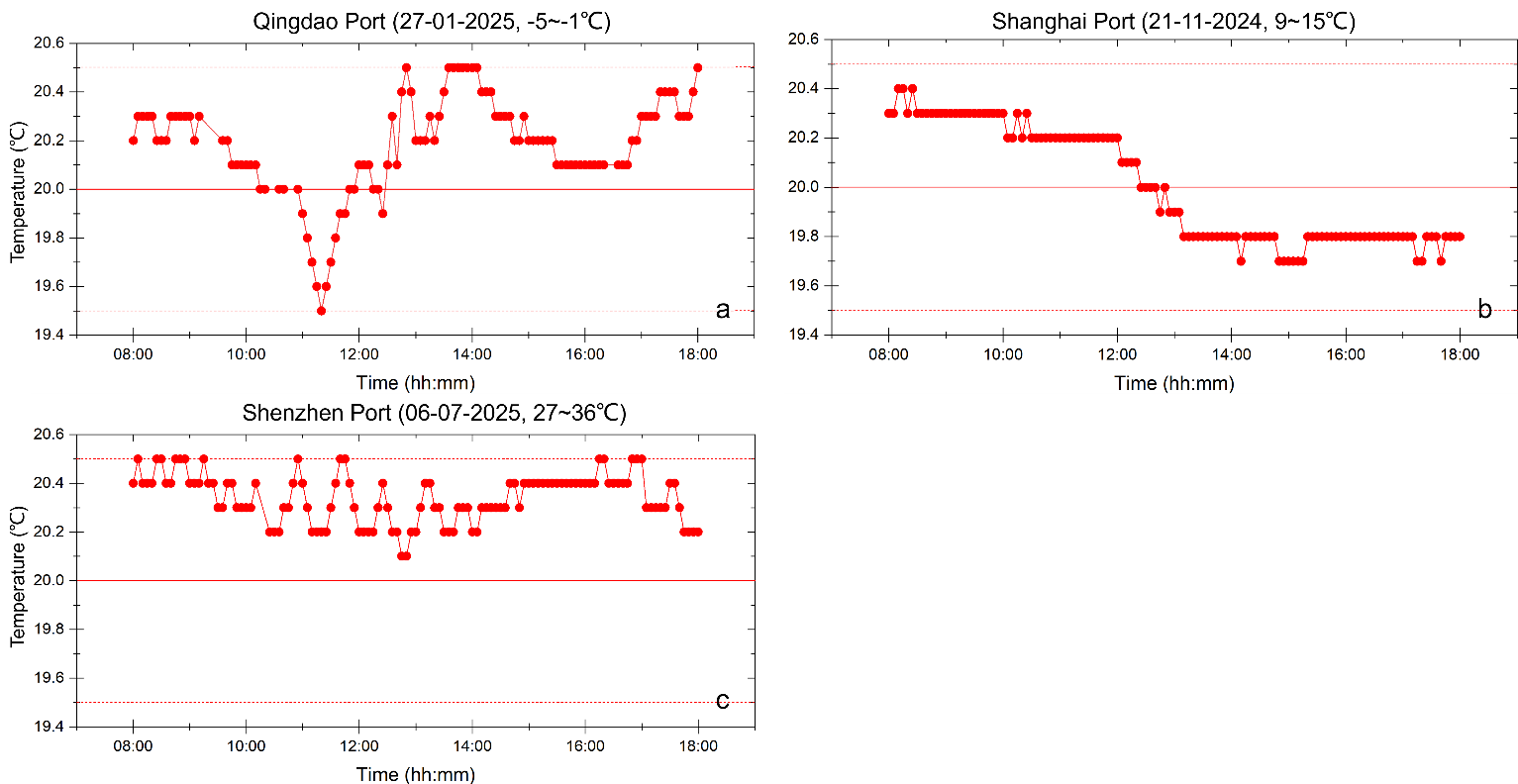


Fig. S2. The spectrometer temperature variations under different ambient temperature conditions. a The spectrometer temperature variations at Qingdao Port at 27 January 2025 with the ambient temperature of -5~-1℃. b The spectrometer temperature variations at Shanghai Port at 21 November 2024 with the ambient temperature of 9~15℃. c The spectrometer temperature variations at Shenzhen Port at 06 July 2024 with the ambient temperature of 27~36℃.


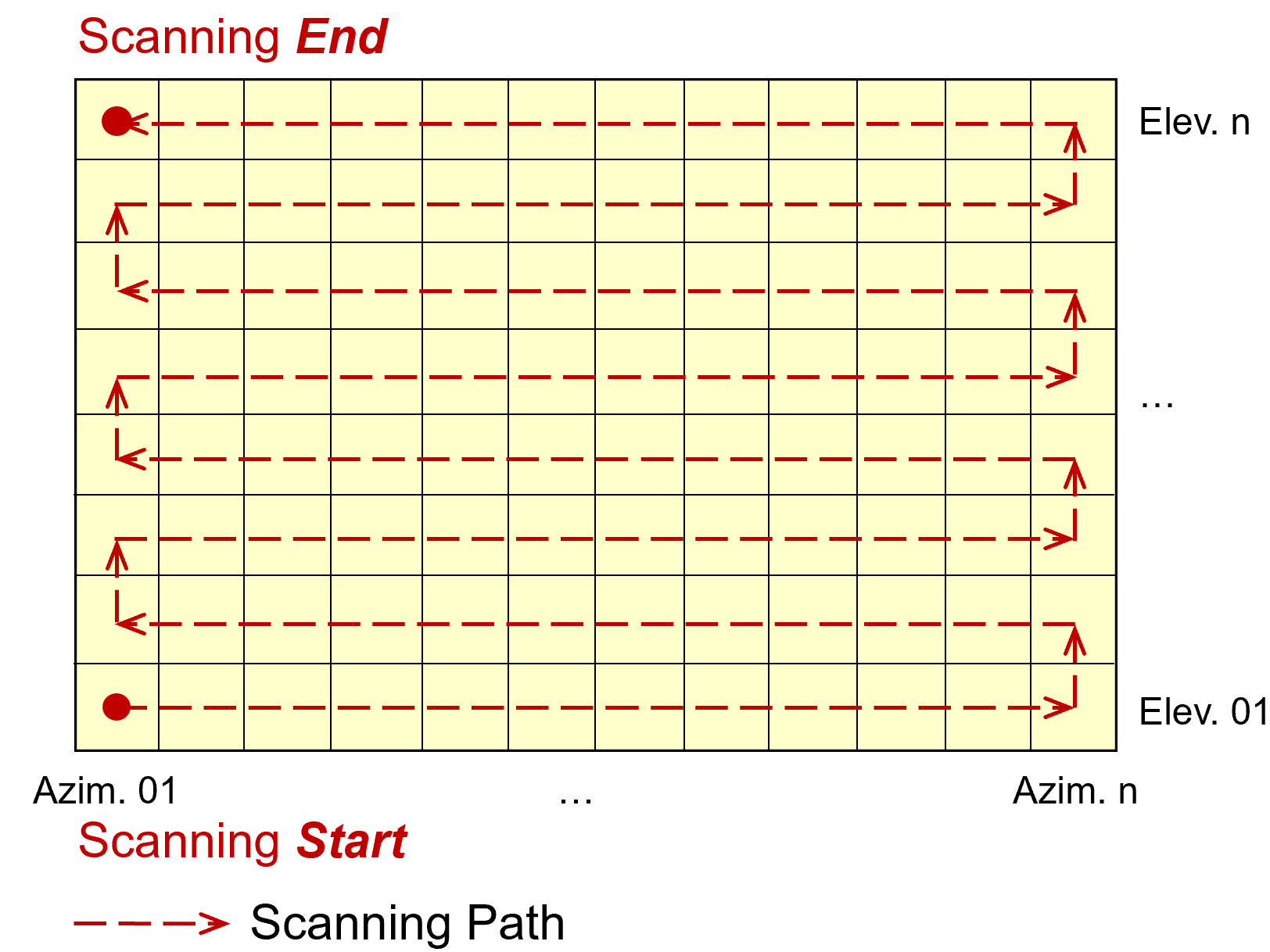


Fig. S3. The observation scheme.


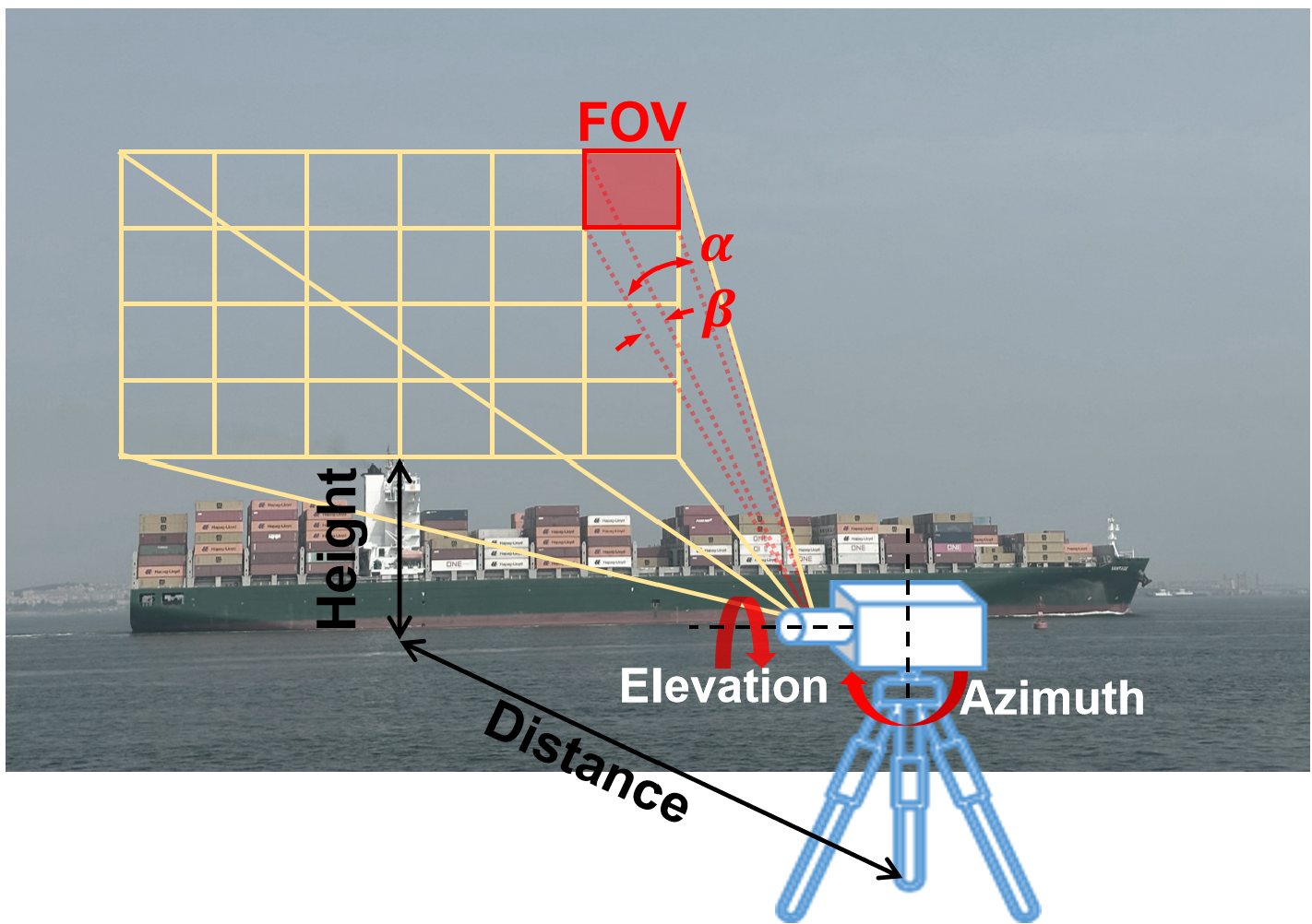


Fig. S4. Schematic of the imaging spatial resolution depending on the field of view (FOV, α×β) of the hyperspectral camera and the distance between the instrument and the emission source.


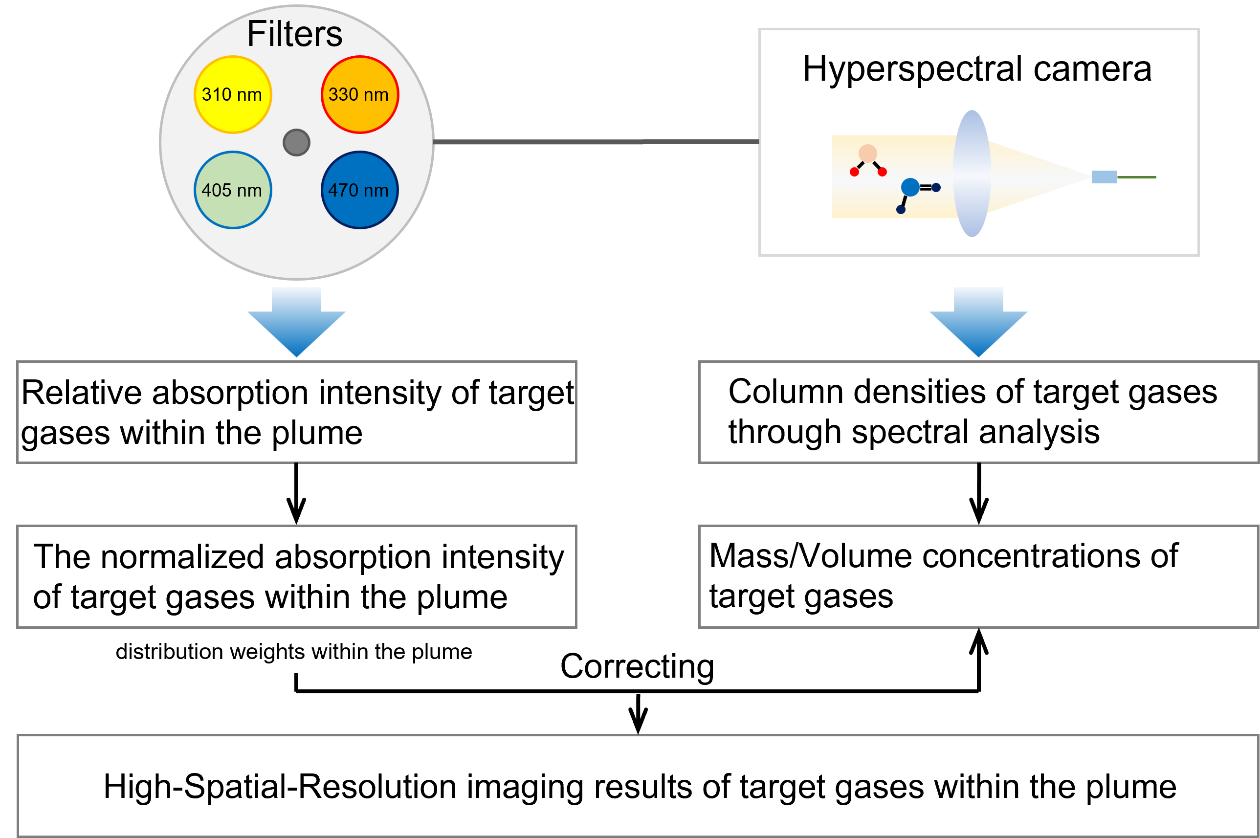


Fig. S5. The flowchart of plume reconstruction.


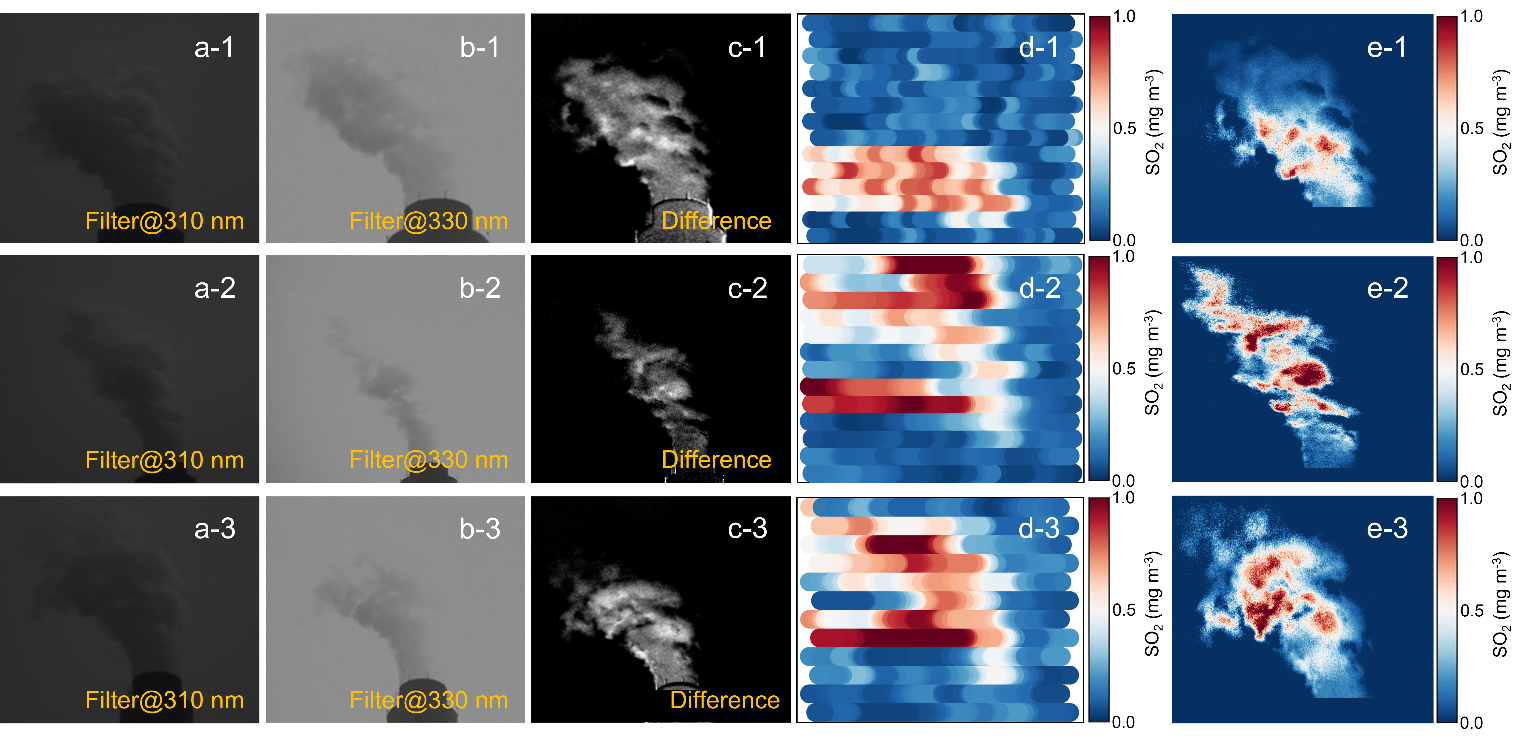


Fig. S6. Reconstruction of plume trace gas concentrations. a Plume signals collected through the filter @310 nm. b Plume signals collected through the filter @330. c Difference in signals between filters @310 nm and @330 nm. d SO_2_ concentrations in the plume measured by the hyperspectral camera. e Reconstruction of the plume with SO_2_ distributions.


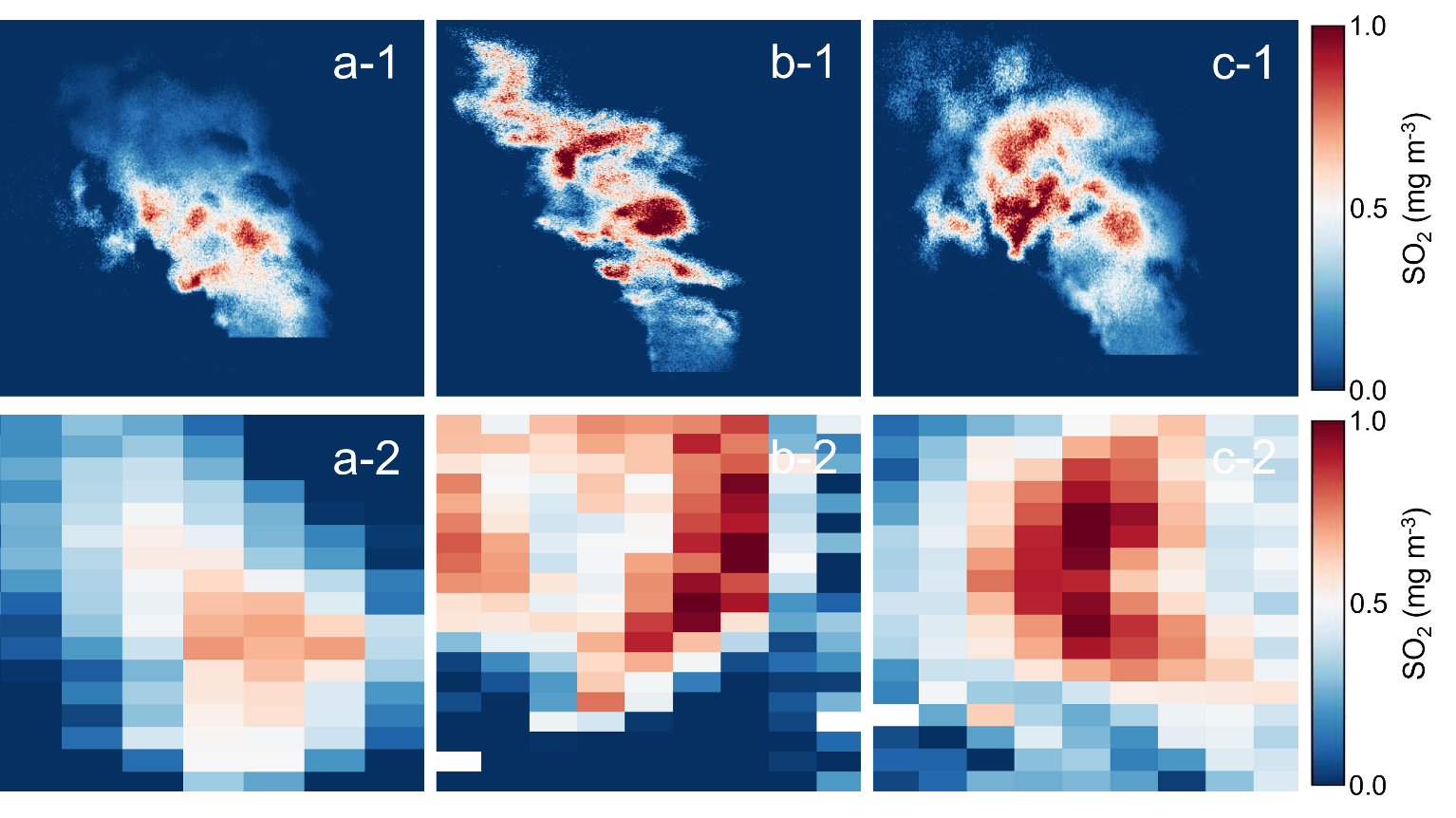


Fig. S7. SO_2_ imaging results. a-1~c-1 SO_2_ imaging results using the fast-hyperspectral imaging remote sensing instrument. a-2~c-2 SO_2_ imaging results using the Imaging DOAS.


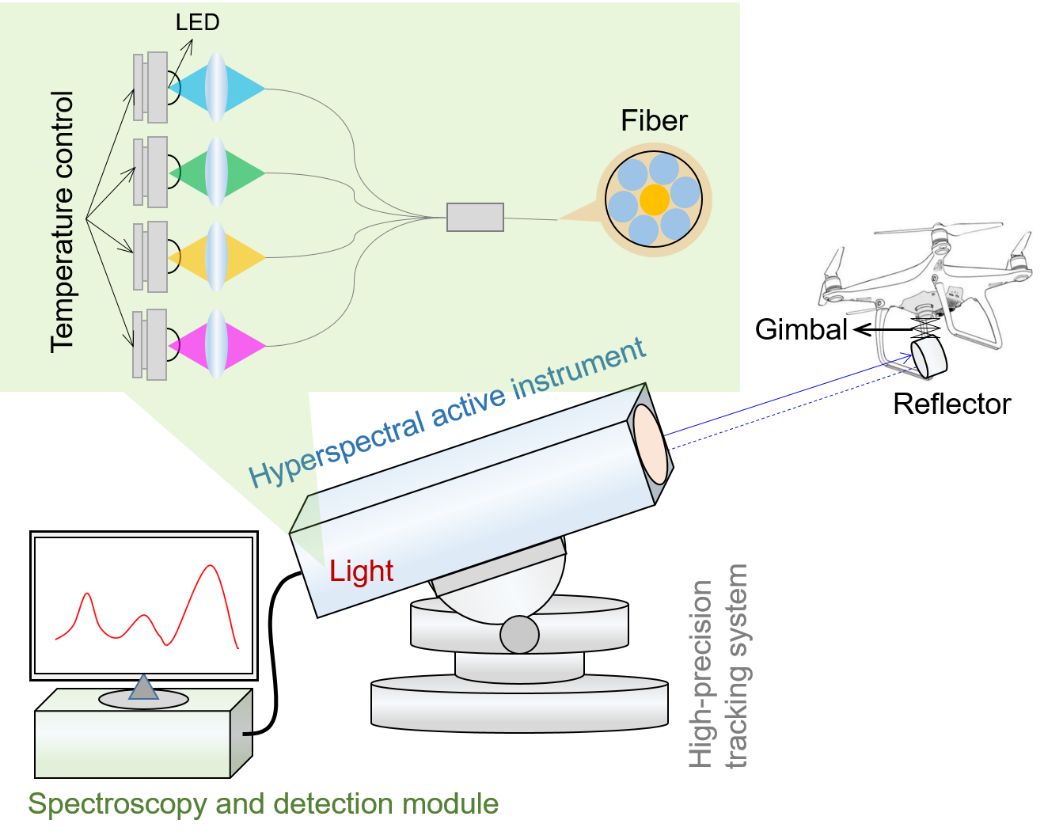


Fig. S8. Nighttime hyperspectral imaging system.


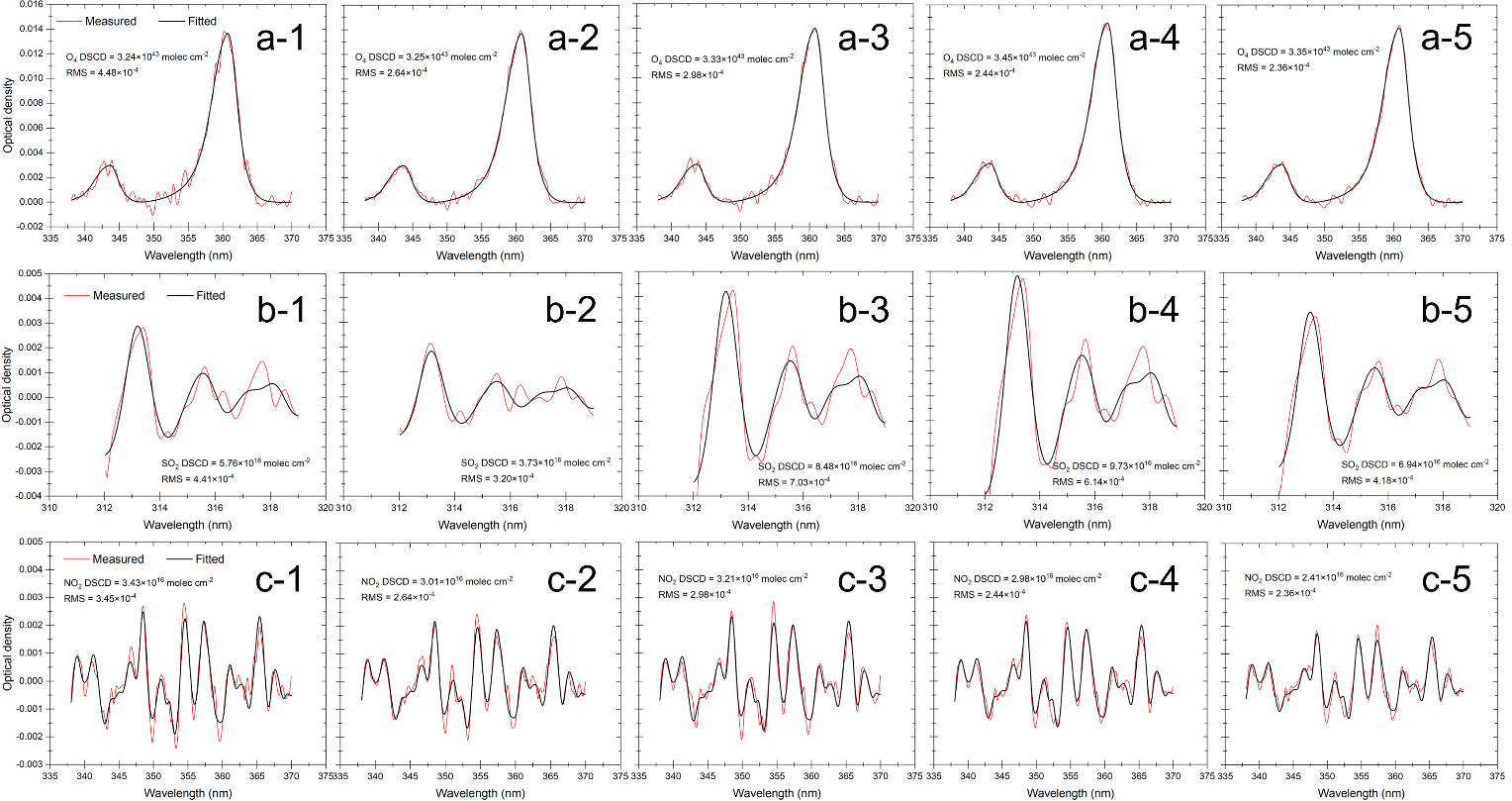


Fig. S9. Typical spectral fitting results. a-c O_4_, SO_2_ and NO_2_ fitting examples. The black and red curves indicate the fitted absorption structures of target species and the derived absorption structures of target species from the measured spectra plus the fit residual, respectively.


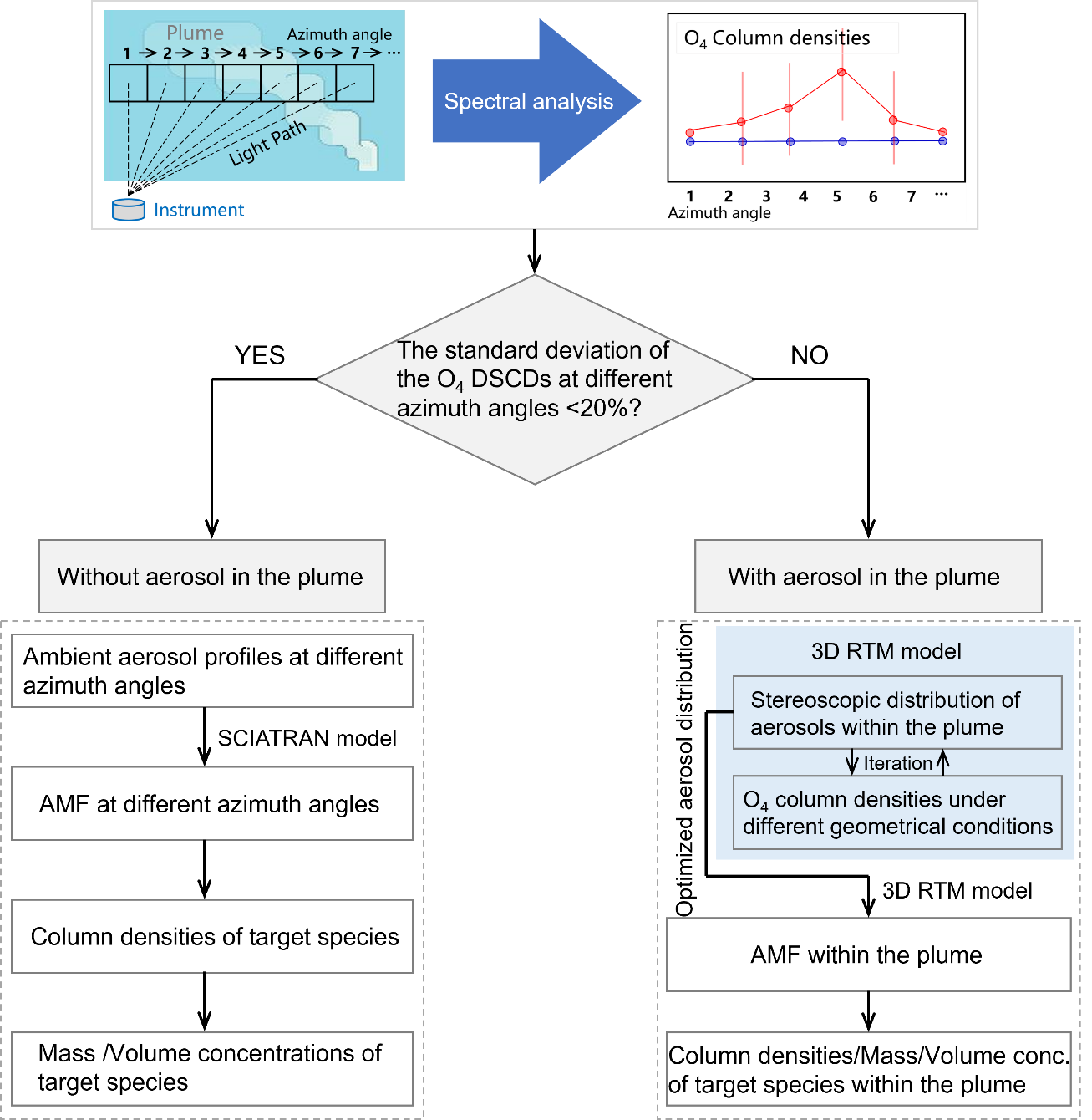


Fig. S10. The flowchart of NO_2_ and SO_2_ concentration calculation.


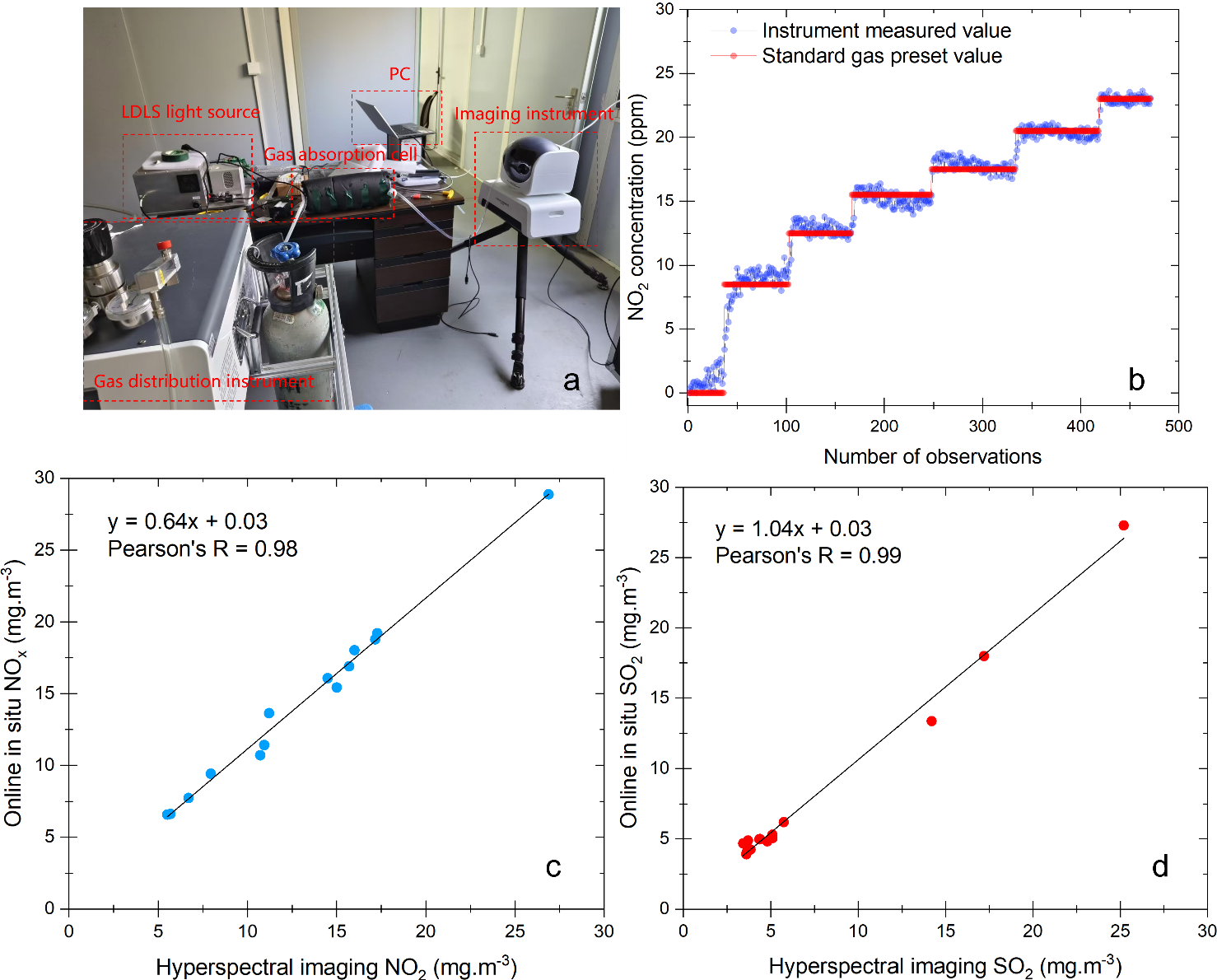


Fig. S11. Data accuracy calibration and verification. a The data calibration system. b The standard gas verification results of NO_2_. c The correlation between NO_2_ imaging results and online in situ measured results from a coal-power plant. d The correlation between SO_2_ imaging results and online in situ measured results from a coal-power plant.


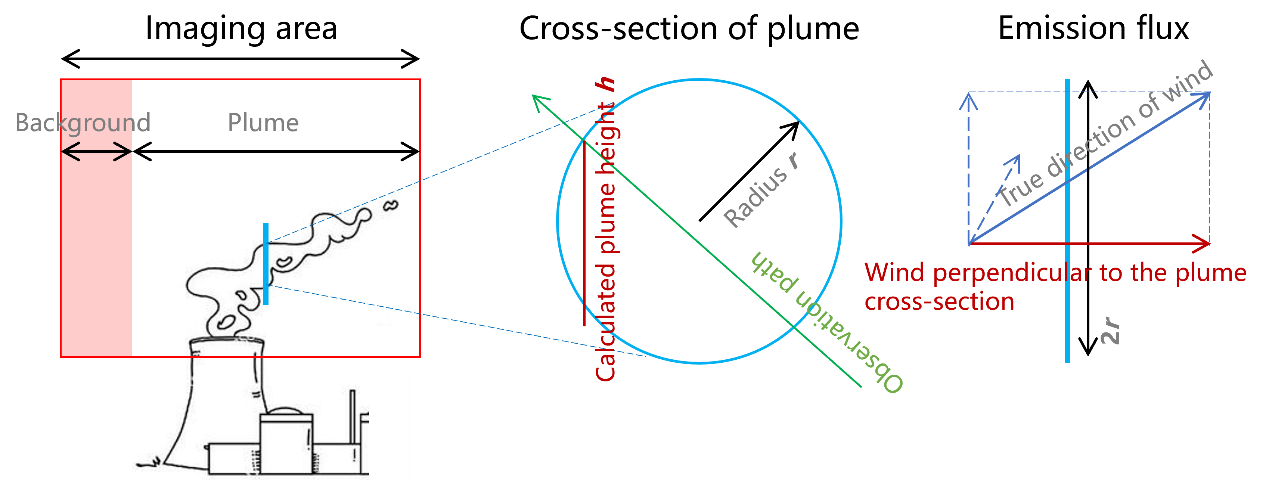


Figure S12. The theoretical diagram of the determination of cross section of the plume and the calculation of the emission fluxes of NO_2_ and SO_2_.

**References**

1. Vandaele A C, Hermans C, Fally S. Fourier transform measurements of SO_2_ absorption cross sections: II.: temperature dependence in the 29000-44000 cm^-1^ (227-345 nm) region. Journal of Quantitative Spectroscopy & Radiative Transfer, 110, 2115-2126, 2009.

2. Wang Y, Lampel J, Xie P, Beirle S, Li A, Wu D, and Wagner T. Ground-based MAX-DOAS observations of tropospheric aerosols, NO_2_, SO_2_ and HCHO in Wuxi, China, from 2011 to 2014, Atmospheric Chemistry and Physics, 17, 2189-2215, 2017.

3. Thalman R M, Volkamer R. Temperature dependent absorption cross-sections of O_2_-O_2_ collision pairs between 340 and 630nm and at atmospherically relevant pressure. Physical Chemistry Chemical Physics, 15, 15371–15381, 2013.

4. Xing C, Liu C, Wang S, Chan K L, Gao Y, Huang X, Su W, Zhang C, Dong Y, Fan G, Zhang T, Chen Z, Hu Q, Su H, Xie Z, and Liu J. Observations of the vertical distributions of summertime atmospheric pollutants and the corresponding ozone production in Shanghai, China. Atmospheric Chemistry and Physics, 17, 14275-14289, 2017.

5. Xing C, Liu C, Lin J, Tan W, Liu T. VOCs hyperspectral imaging: A new insight into evaluate emissions and the corresponding health risk from industries. Journal of Hazardous Materials, 461, 132573, 2024.

6. Zhu Y, Hu Q, Gao M, Zhao C, Zhang C, Liu T, Tian Y, Yan L, Su W, Hong X, Liu C. Quantifying contributions of local emissions and regional transport to NOx in Beijing using TROPOMI constrained WRF-Chem simulation. Remote Sensing, 13, 1798, 2021.
